# Impact of Low-Dose Amino Acid-Chelated Trace Minerals on Performance, Antioxidant Capacity, and Fecal Excretion in Growing-Finishing Pigs

**DOI:** 10.3390/ani15091213

**Published:** 2025-04-24

**Authors:** Yunxia Xiong, Fei Zhao, Yaojie Li, Qiwen Wu, Huaqin Xiao, Shuting Cao, Xuefen Yang, Kaiguo Gao, Zongyong Jiang, Shenglan Hu, Li Wang

**Affiliations:** 1 State Key Laboratory of Swine and Poultry Breeding Industry, Key Laboratory of Animal Nutrition and Feed Science in South China Ministry of Agriculture, Heyuan Branch, Guangdong Laboratory for Lingnan Modern Agriculture, Guangdong Provincial Key Laboratory of Animal Breeding and Nutrition, Institute of Animal Science, Guangdong Academy of Agricultural Sciences, Guangzhou 510640, China; xiongyunxia@gdaas.cn (Y.X.); lyj1714659617@163.com (Y.L.); wuqiwen@gdaas.cn (Q.W.); 18407587188@163.com (H.X.); caoshuting@gdaas.cn (S.C.); yangxuefen@gdaas.cn (X.Y.); gaokaiguo@gdaas.cn (K.G.); jiangzy@gdaas.cn (Z.J.); 2 DeBon Bio-Tech Co., Ltd., Hengyang 421500, China; zhaofei890729@163.com

**Keywords:** amino acid-chelated trace minerals, meat quality, antioxidant capacity, fecal excretion, growing-finishing pigs

## Abstract

Our previous study has shown that replacing 100% inorganic trace minerals with 30% amino acid-chelated ones can enhance antioxidant capacity, improve nutrient digestibility, and reduce fecal excretion in growing-finishing pigs without compromising performance. This study aimed to further reduce the amino acid-chelated trace minerals content in pig diets and assess its impact. Seventy-two growing-finishing barrows (Duroc × Landrace × Yorkshire), with an initial average body weight of 67.04 ± 0.12 kg, were divided into four groups: negative control (NC, no additional trace minerals), high-dose inorganic trace minerals (HITM, 100% inorganic; 75 mg/kg Fe, 10 mg/kg Cu, 65 mg/kg Zn, 25 mg/kg Mn), and two low-dose groups (15 mg/kg Fe, 4 mg/kg Cu, 12.5 mg/kg Zn, 5 mg/kg Mn) receiving either inorganic sulfates (LITM) or amino acid-chelates (LOTM). The trial concluded when the body weight of pigs reached ~130 kg. Results showed that low-dose trace mineral substitution did not adversely affect growth performance, carcass traits, meat quality, or nutrient digestibility in growing-finishing pigs (*p* > 0.05). The LOTM pigs exhibited significantly higher serum glutathione peroxidase, liver total superoxide dismutase (T-SOD), and CuZn-SOD activities, muscle CuZn-SOD and catalase activities, and lower liver malondialdehyde content compared with LITM (*p* < 0.05). Muscle CuZn-SOD in LITM was lower than HITM (*p* < 0.05), but not in LOTM (*p* > 0.05). LOTM showed significantly higher muscle Fe content and lower muscle Mn content compared with HITM (*p* < 0.05), yet its muscle Mn level was higher than that of LITM (*p* < 0.05). Liver Zn content decreased in LITM compared with HITM (*p* < 0.05), but remained unchanged in LOTM (*p* > 0.05). Both LITM and LOTM significantly reduced fecal emissions of Fe, Cu, Zn, and Mn compared with HITM (*p* < 0.05), with greater reductions in Cu, Zn, and Mn in LOTM. In conclusion, low-dose substitution of inorganic or organic trace minerals did not negatively affect growth, carcass traits, meat quality, or nutrient digestibility in growing-finishing pigs, while it effectively reduced fecal heavy metal emissions. Organic trace minerals were more effective in enhancing antioxidant activity and trace mineral deposition.

## Simple Summary

Building on our previous research, which demonstrated that replacing 100% inorganic trace minerals with 30% amino acid-chelated minerals significantly enhanced antioxidant capacity and nutrient utilization efficiency and reduced fecal mineral excretion in growing-finishing pigs while preserving growth performance, this study aimed to investigate the feasibility of implementing an even greater reduction in chelated trace mineral supplementation. We found that low-dose substitution of trace minerals did not have any adverse effects on growth performance, carcass traits, meat quality, or nutrient digestibility. Moreover, the organic trace minerals (amino acid chelates) exhibited greater efficacy in enhancing antioxidant activity, as seen through higher antioxidant enzyme activities and lower malondialdehyde content in the liver. They also showed better trace element deposition in the muscle and liver than inorganic minerals. Additionally, both low-dose inorganic and organic trace mineral substitutions effectively reduced fecal emissions of heavy metals, and low-dose organic ones showed greater efficiency, which is an important aspect considering environmental concerns in the livestock industry. The results of this study are of great significance as they provide new insights into the use of different forms and doses of trace minerals in pig diets.

## 1. Introduction

Trace minerals, including iron, copper, zinc, and manganese, are vital nutrients for animals, crucial for maintaining health, supporting normal growth, and facilitating reproduction [1]. These elements serve as co-factors for numerous enzymes and participate in various biochemical processes vital for life; their deficiency often poses health risks to animals. To meet animals’ nutritional requirements and prevent deficiencies, inorganic or organic trace minerals (OTMs) are commonly supplemented in animal feed. In livestock production, inorganic trace minerals (ITMs), such as sulfates, oxides, chlorides, and carbonates, are frequently used as feed premixes [2]. However, interactions between these ITMs and feed components like fiber, phytic acid, tannic acid, oxalates, and silicates in the gastrointestinal tract can reduce their absorption efficiency and bioavailability [3]. Consequently, trace minerals are often included in animal feed at levels exceeding physiological requirements to optimize production potential and ensure safety margins, leading to substantial excretion in feces and urine and exacerbating soil and surface water pollution [4].

OTMs, formed through the binding of metal elements with proteins, small peptides, amino acids, or organic acids via covalent or ionic bonds, exhibit enhanced stability and reduced reactivity in the digestive tract, facilitating improved intestinal absorption [5,6]. These integrative compounds, including bimetallic or multimetallic complexes, offer a promising approach to enhance trace mineral application in livestock production by reducing dosage while maintaining efficacy, despite their higher cost and superior bioavailability. Notably, current recommended dosages for OTMs in feed are based on outdated research conducted with ITMs, necessitating further investigation [5].

Our previous study demonstrated that a 30% inclusion of composite amino acid-chelated trace minerals (24 mg/kg Fe, 4.5 mg/kg Cu, 19.5 mg/kg Zn, 10.5 mg/kg Mn) effectively sustained performance, carcass characteristics, and meat quality in growing-finishing pigs when used as a reduced replacement for 100% inorganic trace minerals in a corn-soybean meal basal diet [7]. Building on these findings, the present study aims to further optimize the supplementation level of these composite OTMs (15 mg/kg Fe, 4 mg/kg Cu, 12.5 mg/kg Zn, 5 mg/kg Mn) and explore an expanded dosage range for their replacement of ITMs.

## 2. Materials and Methods

### 2.1. Animal Management, Diet, and Experimental Design

A total of 72 growing-finishing barrows (Duroc × Landrace × Yorkshire) with an average initial body weight of 67.04 ± 0.12 kg were allocated into four treatment groups, each consisting of six pens (2.40 m × 1.60 m × 0.9 m) with three pigs per pen. The groups were designated as follows: negative control (NC), high-dose inorganic trace minerals (HITM), low-dose inorganic trace minerals (LITM), and low-dose organic trace minerals (LOTM). The NC group received a corn-soybean basal diet (Table 1) formulated according to NRC (2012) [8] standards without additional trace minerals. The HITM group was fed the basal diet supplemented with 100% ITMs in sulfate form, including 75 mg/kg Fe, 10 mg/kg Cu, 60 mg/kg Zn, and 25 mg/kg Mn. Both the LITM and LOTM groups were supplemented with low-dose trace minerals (15 mg/kg Fe, 4 mg/kg Cu, 12.5 mg/kg Zn, and 5 mg/kg Mn). The LITM group received these minerals in sulfate form, whereas the LOTM group received them as amino acid chelates. The experimental design was shown in Table 2. The amino acid-chelated Fe, Cu, Zn, and Mn were sourced from MINEXO Fe^TM^, MINEXO Cu^TM^, MINEXO Zn^TM^, and MINEXO Mn^TM^ (DeBon Bio-tech Co., Ltd., Hengyang, China), which contained 1.8 × 10^5^ mg/kg Fe, 1.8 × 10^5^ mg/kg Cu, 1.6 × 10^5^ mg/kg Zn, and 1.6 × 10^5^ mg/kg Mn, respectively. The amino acids and metal elements were chelated at a 2:1 molar ratio, employing a mixture of 18 different amino acids—namely, aspartic acid, threonine, serine, glutamic acid, glycine, alanine, valine, isoleucine, leucine, tyrosine, phenylalanine, histidine, lysine, arginine, proline, tryptophan, cystine, and methionine—all of which were derived from hydrolyzed plant proteins. The total amino acid content in the chelates is about 34.65~38.76%. The trace mineral premix of LOTM was formulated by diluting the amino acid chelate trace minerals source with maifanite to align with the experimental design requirements. The concentrations of trace minerals were measured in compliance with China’s National Standard GB/T 13885-2017 [9], and the actual concentrations of these minerals in the experimental diets are displayed in Table 3. The trial lasted for 55 days and concluded when the body weight of pigs reached ~130 kg. Body weights were recorded at the beginning and the end of the experiment, and feed consumption was monitored weekly. All selected experimental animals exhibited good health, normal behavior, and typical food and water intake patterns, along with normal physiological appearance. None of the animals had been exposed to antibiotics prior to or during the experiment. Standard pig management protocols were followed throughout the trial period, with free access to water and feed. The experiment was conducted from February to April in Guangzhou, China. The pigs were housed in open-sided pens with natural ventilation, where the ambient temperature ranged from 14 °C to 27 °C and humidity was approximately 65–85%.

### 2.2. Sampling and Measurements

At the end of the experiment, all pigs were subjected to a 12-h fasting period with ad libitum access to water. Body weights were recorded individually on the following morning prior to sampling. One pig with a body weight closest to the group’s average was selected from each pen for blood sampling and euthanasia. Blood was collected from the anterior vena cava: 1 mL of whole blood was collected in EDTA-K_2_ anticoagulant tubes for hematological assessments, while 10 mL was collected in non-anticoagulant tubes for serum analysis. Serum was isolated by centrifugation at 1509.3× *g* for 10 min at 4 °C. The serum was aliquoted into 1.5 mL centrifuge tubes, immediately snap-frozen in liquid nitrogen, and stored at −80 °C for future analyses. All selected pigs were electrically stunned (250 V, 10 s) and exsanguinated according to standard slaughter procedures. Carcasses were processed through standard abattoir procedures, including removal of the head, hooves, and visceral organs. Samples were collected for the evaluation of carcass traits and meat quality, and the protocol was conducted in accordance with the Industry Standard of the People’s Republic of China (NY/T 821-2019) [17]. The carcasses were split along the dorsal midline, and the left carcasses were used for carcass traits and meat quality analysis. The left hot carcass was weighed to determine the hot carcass weight. Dressing percentage (%) = (Hot carcass weight/Live weight) × 100. The loin-eye area (LA, cm^2^) was measured by tracing the cross-sectional area at the thoracolumbar junction using transparent sulfate paper. The area was calculated using a digital planimeter instrument (KP90N, Koizumi, Osaka, Japan). Backfat thickness was measured at four locations on the left carcass—opposite the first rib, tenth rib, thoracolumbar junction, and lumbar–sacral junction—using a vernier caliper (Shanggong, Shanghai, China). The average backfat thickness value (mm) was calculated. Leaf fat was weighed after being stripped off. The muscle spanning from the fourth rib to the dorsal region near the hip was excised for meat quality analysis. Specifically, tissue samples (approximately 0.5 g each) were aseptically collected from the *longissimus thoracis* muscle and the liver. For the *longissimus thoracis* muscle, samples were taken from the region located between the 9th and 10th ribs in a cranial-to-caudal direction. Additionally, liver tissue samples were collected from the right lower lobe of the liver. These samples were immediately snap-frozen in liquid nitrogen and stored at −80 °C for subsequent analyses.

### 2.3. Meat Quality Assessment

The meat quality analysis was conducted following established methods with slight modifications. Specifically, meat color was evaluated at 45 min, 24 h, and 48 h post-mortem utilizing a portable chromameter (CR-410, Minolta, Chiyoda, Japan) equipped with a D65 light source. pH was determined at 45 min, 24 h, and 48 h postmortem using a portable pH meter (Testo-205, Testo, Lenzkirch, Germany), with triple measurements taken for each time point. A 5 cm × 3 cm × 2 cm strip sample of the *longissimus thoracis* muscle was weighed, suspended via a fishhook in an aerated polyethylene film bag, and stored at 2–4 °C for 24 h and 48 h. After removal from the fishhook, the sample was blotted dry with filter paper and reweighed. Drip loss (%) = (Initial weight − Final weight)/Final weight × 100. Marbling scores were assessed by ten trained evaluators using the National Pork Producers Council (NPPC) standard scoring chart (1991) [18]. Scores ranged from 1 (trace fat) to 5 (excessive fat), with increments of 0.5 allowed between integers. Intramuscular fat was quantified using the Soxhlet extraction method. After trimming fat tissue, approximately 30 g of *longissimus thoracis* muscle was minced, freeze-dried, and powdered. Moisture content (%) = [(Weight before freeze-drying − Weight after freeze-drying)/Weight before freeze-drying] × 100. A 1 g dried meat sample (±0.0001 g) was wrapped in filter paper and placed in a Soxhlet extraction apparatus (SE-A6, Alva, Jinan, China), where it was extracted with n-hexane at 140 °C for 50 min. Following air-drying (10 min) and oven-drying at 102 °C (30 min), the oil-containing aluminum cup was cooled to room temperature and weighed (±0.0001 g). Intramuscular fat (%) = [(Weight of oil-containing cup after extraction − Weight of empty cup)/weight of freeze-dried meat sample] × 100. Shear force was measured using an Instron machine (model 4411, Instron Corp., Norwood, MA, USA) equipped with a Warner–Bratzler blade. The crosshead speed was set to 127 mm/min. The crude protein was determined according to the China National Standard GB/T 6432-2018 [10].

### 2.4. Hematological Determination

A total of 1 mL of blood was collected into an EDTA-K_2_ anticoagulant tube and analyzed within 24 h for hematological parameters using the BC-5000 Vet automated veterinary hematology analyzer (Shenzhen Mindray Bio-Medical Electronics Co., Ltd., Shenzhen, China), which employed a five-part differential methodology.

### 2.5. Serum Biochemical Parameters Measurement

Biochemical analyses were conducted using the VITAL automated analyzer (SELECTRA ProXL, Tokyo, Japan). All assays employed reagent kits supplied by Zhongsheng Beikong Biotechnology Co., Ltd. (Beijing, China), following the manufacturer’s guidelines. The parameters assayed included glucose (GLU), total protein (TP), albumin (ALB), urea (UREA), triglycerides (TG), total cholesterol (TCHO), high-density lipoprotein cholesterol (HDL-C), low-density lipoprotein cholesterol (LDL-C), alanine aminotransferase (ALT), aspartate aminotransferase (AST), total bilirubin (TBILI), alkaline phosphatase (ALP), and creatinine (CRE). Additionally, serum levels of calcium (Ca), phosphorus (P), ceruloplasmin (CP), porcine transferrin (TRF), and haptoglobin (HPT) were determined using commercial kits provided by Nanjing Jiancheng Bioengineering Institute (Nanjing, China), adhering to the provided instructions.

### 2.6. Immunoglobulin Evaluation

Serum concentrations of immunoglobulin A (IgA), immunoglobulin G (IgG), and immunoglobulin M (IgM) were assayed using commercial kits sourced from Jiangsu Meimian Industrial Co., Ltd. (Yancheng, China) in accordance with the manufacturer’s instructions.

### 2.7. Antioxidant Capacity Evaluation

The total antioxidant capacity (T-AOC), malondialdehyde (MDA), catalase (CAT), total superoxide dismutase (T-SOD), Cu/Zn-SOD, Mn-SOD, and glutathione peroxidase (GSH-Px) activities were assayed using commercial kits from Nanjing Jiancheng Bioengineering Institute (Nanjing, China), following the manufacturer’s protocols. Liver and *longissimus thoracis* muscle samples were processed according to a previously described method [7]. Protein content in the homogenates was determined using a BCA kit (Thermo Fisher Scientific, Waltham, MA, USA), and antioxidant activities were normalized per milligram of protein.

### 2.8. Apparent Nutrient Digestibility Detection

Apparent nutrient digestibility was assessed using a method as previously described [7]. Briefly, for three consecutive days prior to the end of the experiment, approximately 300 g of fresh fecal samples were daily collected from each pen, homogenized, dried in an oven at 65 °C for 72 h, and then stored at room temperature for an additional 24 h before grinding. Subsequently, 100 g of the sample was utilized for the determination of energy, dry matter, crude protein, crude ash, and titanium dioxide content. The measurements of dry matter, crude ash, crude protein, and titanium dioxide in both fecal samples and diets adhered to Chinese National Standards GB/T 6435-2014, GB/T 6438-2007, GB/T 6432-2018, and GB 5009.246-2016, respectively [10,13,14,19].

### 2.9. Mineral Analysis

The concentrations of Fe, Cu, Zn, and Mn in serum, liver, muscle, and feces were determined according to the China National Standard (GB 5009.268-2016 [20]). Additionally, the levels of calcium and phosphorus in feces were measured following the methods described in China National Standard GB/T 6436-2018, and GB/T 6437-2018, respectively [15,16].

### 2.10. Statistical Analysis

Data were processed in Excel 2019 (Microsoft Corp., Redmond, DC, USA) and analyzed using IBM SPSS Statistics v18.0 (IBM Corp., Armonk, NY, USA). Results are expressed as mean values with pooled standard errors. For growth performance analyses, the pen served as the experimental unit, whereas individual animals were considered for other parameters. Data normality was assessed using the Shapiro–Wilk test prior to intergroup comparisons. Non-normally distributed variables were analyzed by Kruskal–Wallis one-way ANOVA with false discovery rate (FDR) adjustment for multiple comparisons. Normally distributed data were subjected to one-way ANOVA followed by least significant difference (LSD) post hoc tests. Statistical significance was defined at *p* < 0.05, with 0.05 ≤ *p* < 0.10 indicating a trend toward significance.

## 3. Results

### 3.1. Performance, Carcass Traits, and Meat Quality

As illustrated in Table 4, diets devoid of trace mineral supplementation or replacement of 100% inorganic trace minerals with either low-dose inorganic or organic alternatives showed no significant differences (*p* > 0.05) in growth performance in growing-finishing pigs, including final body weight, average daily gain, average daily feed intake, and feed-to-gain ratio. Similarly, Table 5 demonstrated that these dietary modifications did not significantly affect carcass characteristics (carcass weight, dressing percentage, loin-eye area, average back-fat thickness, or leaf fat weight) compared with HITM (*p* > 0.05). Meat quality analysis (Table 6) indicated non-significant differences (*p* > 0.05) among all treatment groups for meat color, pH, drip loss, shear force, crude protein content, and moisture content. Notably, the NC exhibited significantly lower intramuscular fat content than HITM and LOTM (*p* < 0.05). Furthermore, the LOTM demonstrated superior marbling scores compared with other groups (*p* < 0.05).

### 3.2. Blood Parameters

As shown in Table 7, the NC exhibited a declining trend in EOS count (*p* = 0.067) and HGB levels (*p* = 0.055) versus HITM, whereas low-dose inorganic/organic trace mineral replacements preserved hematological indices within a stable range (*p* > 0.05). Table 8 highlighted notable metabolic adjustments: the NC exhibited significant reductions in TG, CHO, LDL-C, ALP, and CRE levels compared with HITM (*p* < 0.05), accompanied by elevated HPT levels (*p* < 0.05). Substituting 100% inorganic trace minerals with low-dose inorganic or organic counterparts significantly increased serum ALB (*p* < 0.05) and further decreased TG, LDL-C, ALP, and CRE levels (*p* < 0.05). Both the devoid of additional trace minerals and the aforementioned substitution were associated with an increase in serum ALT activity (*p* < 0.05). Notably, the LOTM exhibited significantly higher serum CRE levels compared with the LITM (*p* < 0.05) and lower Ca and HPT levels (*p* < 0.05). The low-dose mineral-supplemented groups exhibited significantly higher IgG and IgM levels than NC (*p* < 0.05, Table 9). However, HITM showed no significant changes in the levels of IgA, IgG, and IgM when compared with other groups (*p* > 0.05, Table 9).

### 3.3. Antioxidant Capacity

**Figure 1:**
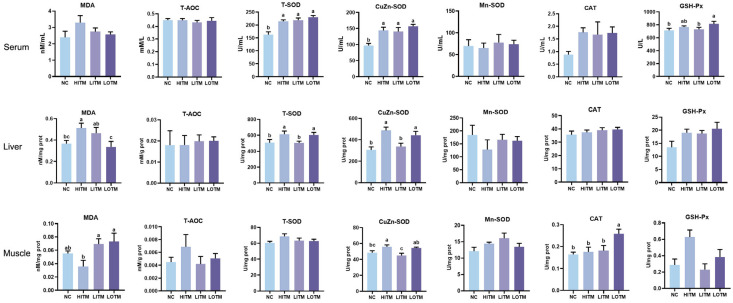
Effects of dietary replacement of high-dose inorganic trace minerals (Fe, Cu, Zn, Mn) with low-dose inorganic/organic alternatives on the antioxidant profiles in growing-finishing pigs. Data represent mean ± SEM (*n* = 6). ^abc^ Columns with different superscripts differ significantly (one-way ANOVA followed by LSD test, *p* < 0.05). Abbreviations: NC, negative control; HITM, high-dose inorganic trace minerals; LITM, low-dose inorganic trace minerals; LOTM, low-dose organic trace minerals; MDA, malondialdehyde; T-AOC, total antioxidant capacity; T-SOD, total superoxide dismutase; CuZn-SOD, copper-zinc superoxide dismutase; Mn-SOD, manganese superoxide dismutase; CAT, catalase; GSH-px, glutathione peroxidase; prot, protein.

As depicted in Figure 1, the NC exhibited significant reductions in serum T-SOD and CuZn-SOD activities compared with the HIMT, as well as hepatic MDA content, T-SOD, and CuZn-SOD activities (*p* < 0.05). Additionally, a decreasing trend was observed in serum CAT activity (*p* = 0.083). Both the NC and the LITM displayed a markable reduction of muscle CuZn-SOD activity compared with the HIMT (*p* < 0.05), whereas the LOTM showed no statistically significant alteration (*p* > 0.05). Notably, the LOTM demonstrated significantly higher activities of serum GSH-Px, hepatic T-SOD and CuZn-SOD, muscle CuZn-SOD, and CAT (*p* < 0.05) compared with the LITM. Furthermore, a significant reduction in hepatic MDA content was also observed in the LOTM compared with HITM and LITM (*p* < 0.05).

### 3.4. Apparent Nutrient Digestibility

As shown in Table 10, substituting 100% of inorganic trace minerals in the diet with either low-dose inorganic or organic alternatives did not result in any significant variation in the apparent digestibility of energy, dry matter, crude protein, and crude ash (*p* > 0.05). However, when compared with the negative control (NC), a notable improvement in apparent digestibility of energy, dry matter, crude protein, and crude ash was observed (*p* < 0.05).

### 3.5. Minerals Deposited in Tissues

As shown in Table 11, the serum Zn content was increased in both the LITM and LOTM compared with the NC (*p* < 0.05). Hepatic concentrations of Fe, Cu, and Zn were markedly higher in both the LOTM and HITM compared with the NC (*p* < 0.05). However, no significant difference in hepatic trace mineral deposition was observed in the LITM compared with the NC (*p* > 0.05). Notably, the muscle Fe content was significantly elevated in the LOTM compared with the other three groups (*p* < 0.05). In contrast, the muscle Mn content was significantly lower in both the NC and the low-dose groups compared with HITM (*p* < 0.05). Moreover, in low-dose groups, the muscle Mn content was significantly higher than the NC (*p* < 0.05), with the LOTM exhibiting a much higher concentration than the LITM (*p* < 0.05).

### 3.6. Fecal Excretion

**Figure 2:**
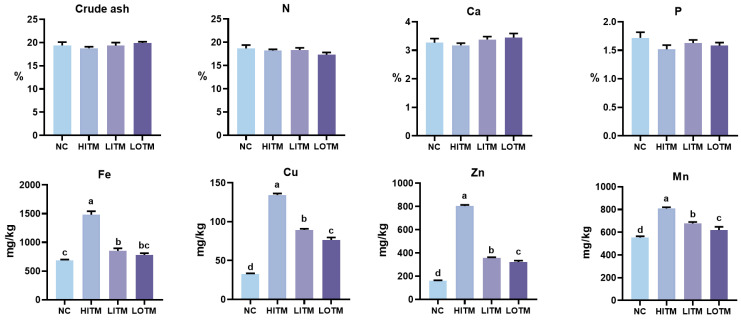
Effects of dietary replacement of high-dose inorganic trace minerals (Fe, Cu, Zn, Mn) with low-dose inorganic/organic alternatives on the fecal excretion in growing-finishing pigs. Data represent mean ± SEM (*n* = 6). ^abcd^ Columns with different superscripts differ significantly (one-way ANOVA followed by LSD test, *p* < 0.05). Abbreviations: NC, negative control; HITM, high-dose inorganic trace minerals; LITM, low-dose inorganic trace minerals; LOTM, low-dose organic trace minerals; N, nitrogen; Ca, calcium; P, phosphorus.

As illustrated in Figure 2, all treatments had no effect on the fecal emissions of crude ash, nitrogen, calcium, or phosphorus (*p* > 0.05). The fecal excretion levels of Fe, Cu, Zn, and Mn were significantly decreased in both the NC and the low-dose treatment groups compared with the HITM (*p* < 0.05). Furthermore, the fecal excretion of Cu, Zn, and Mn in the LOTM was significantly lower than that in the LITM, with reductions of 14.34%, 10.15%, and 8.70%, respectively (*p* < 0.05).

## 4. Discussion

The efficacy of OTM substitution appears contingent upon species-specific requirements and inherent mineral profiles within the basal diet. Chen et al. [21] reported that replacing inorganic trace minerals with 20% or 40% hydroxy methionine chelates significantly increased the ADG in growing-finishing pigs. This aligns with a broiler study showing that replacing 1000 mg/kg inorganic trace minerals with 300 or 500 mg/kg organic trace minerals significantly boosted ADG during both 22~53 d and entire growth phases (1~53 d) [22]. Partial substitution strategies indicated superior OTM bioavailability, as growth parameters remained unaffected when approximately 30% OTM replaced 100% ITM in weaned piglets [23] and growing-finishing pigs [7]. Further reducing the OTM dose to levels employed in this study had no adverse impact on performance, suggesting potential for substantial mineral input reduction without compromising productivity. However, paradoxically, equivalent feed efficiency improvements were observed between ITM and protein-chelated forms in piglets receiving 50%~100% mineral supplementation [24]. This phenomenon likely originates from inherent mineral sufficiency in the basal diet [25,26,27,28]. According to NRC (2012) [8] requirements, the basal diet (NC) in this study already exceeded standards for Fe (70.7 vs. 41.9 mg/kg), Cu (3.29 vs. 3.09 mg/kg), and Mn (57.4 vs. 2 mg/kg). Notably, Zn constituted the sole marginally deficient element, with NC (22.1 mg/kg), LITM (34.68 mg/kg), and LOTM (34.61 mg/kg) groups all below the 50 mg/kg requirement. These findings indicate that OTM’s bioavailability advantages become physiologically relevant primarily when addressing specific mineral deficits rather than meeting baseline requirements. Moreover, the supplementation strategy for trace minerals should preferably be based on the content of trace elements in the basal diet.

The dose-dependent effects of OTM substitution on carcass characteristics and meat quality are increasingly evident across species. Wang et al. [29] observed linear fat thickness reduction with graded methionine hydroxy analogue chelate replacement (20%~100% ITM). While a 70% OTM replacement has demonstrated carcass trait improvements in a prior study [30], our findings showed low-dose substitution (lower than 30% OTM) maintained performance without adverse effects. Notably, the absence of significant benefits beyond marbling score enhancement may reflect insufficient zinc supplementation (34.6 mg/kg vs. NRC-recommended 50 mg/kg) in our trial. Copper substitution studies reveal mechanistic insights: Zhao et al. [31] identified 80 mg/kg copper methionine as optimal, increasing growth hormone secretion and loin depth versus sulfate controls. Complementary research [32] demonstrates zinc amino acid chelates (especially Zn-Met) enhance myoblast proliferation and differentiation at the cellular level. These endocrine and cellular mechanisms may synergistically improve lean deposition. OTM’s antioxidant capacity significantly enhances meat preservation across production systems. Jiang et al. [33] reported peptide-chelated substitutions reduced pork lipid oxidation and drip loss via SOD activation. Remarkably, one-third OTM dosage achieved similar efficacy, corroborated by poultry data showing pH_45min_ elevation and 48 h drip loss reduction [34]. Natalello et al. [35] further validated this using zinc glycinate, reducing chilling loss. Paradoxically, flavor-related amino acids (methionine, phenylalanine, arginine) decreased despite technical improvements [29]. These findings highlight the need to balance technological meat quality parameters with organoleptic properties through optimized OTM formulations.

Blood biomarkers provide critical insights into livestock performance and metabolic regulation. For instance, serum total protein and albumin levels serve dual physiological functions: reflecting proteostatic balance (absorption/synthesis/catabolism) and indicating immunological competence [36]. Our prior work demonstrated that 100% replacement of ITM with reduced-dose OTM elevated the serum triglyceride, transferrin, and calcium contents [7]. This aligns with broiler data showing higher alkaline phosphatase activity with 300 mg/kg OTM versus 1000 mg/kg ITM [34]. The current study revealed distinct metabolic impacts of mineral supplementation strategies. Both low-dose substitutions (ITM/OTM) enhanced serum albumin while further reducing triglycerides, low-density lipoprotein cholesterol, alkaline phosphatase, and creatinine levels compared with the HITM. Trace mineral-deficient diets exacerbated metabolic suppression, decreasing triglycerides, cholesterol, low-density lipoprotein cholesterol, alkaline phosphatase, and creatinine relative to the HITM. Immunoglobulin dynamics revealed zinc-dependent immune modulation. IgG is activated in long-term immunity, IgM is the first antibody produced during infection, and IgA bridges humoral/mucosal defenses [37]. Zhang et al. [23] reported IgG elevation with one-third OTM substitution in piglets. However, in this study, we found that the devoid of trace minerals in supplementation resulted in a decrease in the levels of serum IgG and IgM, potentially attributable to zinc deficiency impairing lymphocyte maturation.

The antioxidant predominance of organic trace minerals over inorganic counterparts in swine nutrition is well substantiated. Our findings corroborate this superiority, demonstrating higher antioxidant capacity in the OTM-supplemented group versus the ITM at an equivalent dose. Ma et al. [38] demonstrated that iron glycine chelate supplementation increased serum SOD levels while decreasing MDA levels in weaned piglets, correlating with improved growth performance and lower diarrhea incidence through enhanced iron transport efficiency and gut microbiota modulation. Additionally, copper-specific studies showed that 30~60 mg/kg copper citrate supplementation elevated serum Cu/Zn-SOD levels while reducing lipid peroxidation [39]. Systemic antioxidant enhancement was further evidenced by Liu et al. [40], where complete OTM substitution (protein-chelated Fe/Cu/Mn/Zn) increased hepatic Cu/Zn-SOD and GSH-Px in finishing pigs, outperforming inorganic formulations. Mechanistic insights from Tang et al. [41] identified zinc lactate’s capacity to activate the AMPK-Nrf2-p62 signaling pathway in jejunal epithelium, enhancing mitochondrial complex I activity and cellular antioxidant capacity compared with zinc sulfate. Dose-response analyses [34] delineated optimal OTM inclusion: 300 mg/kg elevated plasma CAT and CuZn-SOD, while 500 mg/kg maximized hepatic GSH-Px activity. Conversely, we found that trace mineral-deficient regimens significantly compromised antioxidant defenses, underscoring the essentiality of balanced mineral supplementation.

The bioavailability and metabolic regulation of OTM have been systematically validated across species. Comparative analyses demonstrate OTM’s superior mineral retention efficiency versus ITM, with higher serum Fe, Cu, and Mn concentrations in piglets supplemented with OTM over 4 weeks, alongside enhanced hepatic Fe and bone Zn/Mn deposition [42]. This retention advantage translates to reduced environmental impact, as evidenced by lower fecal excretion of Cu, Zn, Fe, and Mn in weaned piglets receiving one-third OTM substitution [23] and decreased metal excretion in broilers fed 300~500 mg/kg OTM versus 1000 mg/kg ITM [22,34]. Mechanistically, OTM’s absorption superiority stems from two synergistic properties. First, chemical stability: chelation with ligands like glycine or small peptides prevents insoluble complex formation with phytates, increasing intestinal solubility versus sulfates [43,44]. Then, targeted delivery: amino acid-chelated Mn (20~60 ppm) preferentially accumulates in functional corpora lutea [45], while small peptide-chelated iron (75~100 mg/kg) enhances hepatic/kidney Fe deposition through duodenal DMT1 downregulation [46]. Notably, glycine-chelated metals exhibit higher apparent digestibility than sulfate forms through minimized gastrointestinal interference [47]. Our findings corroborate this mechanism, demonstrating that low-dose OTM supplementation reduces fecal heavy metal excretion while enhancing tissue mineral deposition capacity, suggesting a dual advantage of precision nutrition and environmental sustainability.

In the field of research centered on amino acid chelates of trace minerals, researchers frequently neglect the augmentation in amino acid content within these chelates when incorporated into diets. Our current investigation has unveiled a positive impact on finishing pigs when dietary levels of amino acid-chelated trace minerals are reduced. This effect seems to arise from the combined influence of trace minerals and the elevated amino acid content. Based on our calculated data, the total amino acid content in the diet of the LOTM group exceeds that of the other experimental groups by 0.74 to 0.83 mg per 100 kg of diet. Although the disparity in the total amino acid content is exceedingly minute, it is imperative to consider the balance of amino acids between amino acid-chelated trace minerals and inorganic trace minerals in future research.

## 5. Conclusions

Substituting 100% inorganic trace minerals with low-dose inorganic or organic trace minerals had no adverse effects on growth performance, meat quality, or apparent nutrient digestibility in growing-finishing pigs. Amino acid chelated trace minerals exhibited greater efficiency compared with inorganic ones in augmenting antioxidant activity, facilitating tissue accumulation of trace minerals, and decreasing heavy metal excretion in feces. Further research is needed to determine the lower limit for the extremely low-level addition of organic trace minerals to the diets of growing-finishing pigs based on this foundation.

## Figures and Tables

**Table 1:** Components and nutrient levels of the basal diet (air-dry basis).

Ingredients	Content, %	Nutrient Levels ^b^	Content
Corn	79.67	Digestive energy, kcal/kg	3050.03
Soybean meal (46%)	15.33	Crude protein, %	15.00
Soybean oil	1.39	Crude fiber, %	2.22
CaHPO_4_	0.90	Crude fat, %	6.37
Limestone	0.95	Ash, %	4.44
NaCl	0.50	Ca, %	0.69
Choline chloride (50%)	0.10	Total phosphorus, %	0.48
L-Lysine hydrochloride	0.21	SID Lys, %	0.83
DL-Methionine	0.02	SID Met + Cys, %	0.49
L-Tryptophan	0.03	SID Thr, %	0.50
Trace minerals Premix	0.10	SID Trp, %	0.17
Other Premix ^a^	0.80		
Total	100		

^a^ The premix provided the following per kg of diets: VA 12,400 U, VD_3_ 2800 U, VE 30 U, VK 5 mg, VB_12_ 40 μg, VB_1_ 3 mg, VB_2_ 10 mg, nicotinic acid 40 mg, D-pantothenic acid 15 mg, folic acid 1 mg, VB_6_ 8 mg, biotin 0.08 mg, CaI_2_O_6_ 0.92 mg, and Na_2_SeO_3_ 0.77 mg. ^b^ Nutrient levels were measured values, except that the digestible energy, SID Lys, SID Met, SID Thr, and SID Trp were calculated values. The crude protein, crude fiber, crude fat, moisture, ash, Ca, and total phosphorus were all determined according to the China National Standard, and the document numbers are GB/T 6432-2018, GB/T 6434-2022, GB/T 6433-2006, GB/T 6435-2014, GB/T 6438-2007, GB/T 6436-2018, and GB/T 6437-2018, respectively [10,11,12,13,14,15,16].

**Table 2:** Experimental design.

Items	Forms	NC	HITM	LITM	LOTM
Fe, mg/kg	Amino acid chelates	-	-	-	15
	Sulphates	-	75	15	-
Cu, mg/kg	Amino acid chelates	-	-	-	4
	Sulphates	-	10	4	-
Zn, mg/kg	Amino acid chelates	-	-	-	12.5
	Sulphates	-	65	12.5	-
Mn, mg/kg	Amino acid chelates	-	-	-	5
	Sulphates	-	25	5	-

Abbreviations: NC, negative control; HITM, high-dose inorganic trace minerals; LITM, low-dose inorganic trace minerals; LOTM, low-dose organic trace minerals.

**Table 3:** Measured values of the trace minerals in experimental diets (air-dry basis).

Items	NC	HITM	LITM	LOTM
Fe, mg/kg	70.70	145.79	87.29	87.74
Cu, mg/kg	3.29	13.44	7.68	8.67
Zn, mg/kg	22.10	82.16	34.68	34.61
Mn, mg/kg	57.40	82.57	62.83	63.77

Abbreviations: NC, negative control; HITM, high-dose inorganic trace minerals; LITM, low-dose inorganic trace minerals; LOTM, low-dose organic trace minerals. The concentrations of trace minerals were determined according to the China National Standard GB/T 13885-2017 [9]. The four-subsample sampling approach was employed, with three samples collected per group. Mean values are presented (*n* = 3).

**Table 4:** Effects of dietary replacement of high-dose inorganic trace minerals (Fe, Cu, Zn, Mn) with low-dose inorganic/organic alternatives on growth performance in growing-finishing pigs.

Items	NC	HITM	LITM	LOTM	SEM	*p*-Value
Initial BW, kg	67.00	67.10	66.89	67.17	0.119	0.873
Final BW, kg	132.27	134.01	132.60	131.26	0.724	0.632
ADG, kg/d	1.19	1.22	1.19	1.17	0.013	0.570
ADFI, kg/d	3.54	3.53	3.56	3.44	0.030	0.572
F/G	2.98	2.91	2.98	2.96	0.024	0.694

Values are presented as mean and pooled SEM, *n* = 6. A pen is an analysis unit. Data in the same row with no or the same letter indicate no significant difference (*p* > 0.05), while different letters mean significant difference (*p* < 0.05). Abbreviations: NC, negative control; HITM, high-dose inorganic trace minerals; LITM, low-dose inorganic trace minerals; LOTM, low-dose organic trace minerals; BW, body weight; ADG, average daily gain; ADFI, average daily feed intake; F/G, feed-to-gain ratio.

**Table 5:** Effects of dietary replacement of high-dose inorganic trace minerals (Fe, Cu, Zn, Mn) with low-dose inorganic/organic alternatives on carcass traits in growing-finishing pigs.

Items	NC	HITM	LITM	LOTM	SEM	*p*-Value
Carcass weight, kg	100.73	95.12	96.22	96.44	1.056	0.262
Dressing percentage, %	75.17	74.12	74.15	74.13	0.355	0.692
Loin-eye area, cm^2^	48.21	50.68	46.51	48.16	2.075	0.927
Average back-fat thickness, mm	30.50	27.79	31.17	33.25	0.926	0.222
Leaf fat weight, kg	1.87	1.77	1.73	2.10	0.086	0.447

Values are presented as mean and pooled SEM, *n* = 6. Data in the same row with no or the same letter indicate no significant difference (*p* > 0.05), while different letters mean significant difference (*p* < 0.05). Abbreviations: NC, negative control; HITM, high-dose inorganic trace minerals; LITM, low-dose inorganic trace minerals; LOTM, low-dose organic trace minerals.

**Table 6:** Effects of dietary replacement of high-dose inorganic trace minerals (Fe, Cu, Zn, Mn) with low-dose inorganic/organic alternatives on meat quality traits in growing-finishing pigs.

Items	NC	HITM	LITM	LOTM	SEM	*p*-Value
Meat color						
L*_45min_	41.75	42.32	42.44	41.49	0.292	0.637
a*_45min_	13.36	13.35	13.19	13.28	0.139	0.975
b*_45min_	4.68	4.96	4.79	4.79	0.106	0.836
L*_24h_	52.43	52.15	52.42	51.52	0.516	0.926
a*_24h_	14.09	14.19	14.00	13.88	0.129	0.871
b*_24h_	6.66	6.62	6.57	6.40	0.130	0.913
L*_48h_	52.95	53.03	52.88	52.14	0.463	0.911
a*_48h_	14.62	14.87	14.87	14.45	0.193	0.854
b*_48h_	6.67	7.01	6.70	6.66	0.101	0.582
pH						
pH_45min_	6.54	6.50	6.53	6.56	0.028	0.922
pH_24h_	5.48	5.49	5.48	5.52	0.016	0.805
pH_48h_	5.43	5.48	5.45	5.50	0.016	0.448
Drip loss						
Drip loss_24h_, %	2.23	2.21	2.29	2.34	0.062	0.892
Drip loss_48h_, %	2.79	2.48	2.76	2.71	0.082	0.555
Shear force, N	51.50	47.74	49.10	49.16	1.623	0.890
Marbling score	2.42 ^b^	2.65 ^b^	2.65 ^b^	3.28 ^a^	0.108	0.020
Intramuscular fat, %	1.88 ^b^	2.83 ^a^	2.35 ^ab^	3.01 ^a^	0.146	0.027
Crude protein, %	22.10	22.16	21.84	21.83	0.109	0.621
Moisture, %	73.38	72.80	72.95	73.07	0.166	0.661

Values are presented as mean and pooled SEM, *n* = 6. Data in the same row with no or the same letter indicate no significant difference (*p* > 0.05), while different letters mean significant difference (*p* < 0.05). Abbreviations: NC, negative control; HITM, high-dose inorganic trace minerals; LITM, low-dose inorganic trace minerals; LOTM, low-dose organic trace minerals; L*, luminosity; a*, redness; b*, yellowness.

**Table 7:** Effects of dietary replacement of high-dose inorganic trace minerals (Fe, Cu, Zn, Mn) with low-dose inorganic/organic alternatives on hematology in growing-finishing pigs.

Items	NC	HITM	LITM	LOTM	SEM	*p*-Value
WBC, ×10^9^/L	15.50	18.63	15.12	17.51	0.702	0.245
NEU, ×10^9^/L	2.61	4.91	4.00	4.18	0.412	0.258
LYM, ×10^9^/L	12.47	12.95	10.26	12.31	0.554	0.342
MON, ×10^9^/L	0.15	0.27	0.27	0.30	0.025	0.167
EOS, ×10^9^/L	0.17	0.43	0.54	0.65	0.068	0.067
BAS, ×10^9^/L	0.10	0.07	0.05	0.08	0.007	0.107
RBC, ×10^12^/L	7.04	7.22	7.33	7.44	0.097	0.521
HGB, g/L	126.00	133.33	133.00	138.67	1.685	0.055
HCT, %	39.43	40.90	40.90	42.57	0.516	0.204
PLT, ×10^9^/L	307.00	296.67	264.67	313.33	12.912	0.580

Values are presented as mean and pooled SEM, *n* = 6. Data in the same row with no or the same letter indicate no significant difference (*p* > 0.05), while different letters mean significant difference (*p* < 0.05). Abbreviations: NC, negative control; HITM, high-dose inorganic trace minerals; LITM, low-dose inorganic trace minerals; LOTM, low-dose organic trace minerals; WBC, white blood cells; NEU, neutrophile granulocyte; LYM, lymphocyte; MON, monocytes; EOS, eosinophilic granulocyte; BAS, basophilic granulocyte; RBC, red blood cells; HGB, hemoglobin; HCT, hematocrit; PLT, platelets.

**Table 8:** Effects of dietary replacement with high-dose inorganic trace minerals (Fe, Cu, Zn, Mn) with low-dose inorganic/organic alternatives on serum biochemical parameters in growing-finishing pigs.

Items	NC	HITM	LITM	LOTM	SEM	*p*-Value
GLU, mM/L	4.17	3.96	3.67	3.62	0.129	0.406
TP, g/L	56.47	56.25	54.87	56.17	0.733	0.882
ALB, g/L	28.50 ^b^	28.18 ^b^	30.04 ^a^	30.62 ^a^	0.314	0.005
URE, mM/L	3.66 ^b^	3.88 ^ab^	4.44 ^a^	4.59 ^a^	0.134	0.028
TG, mM/L	0.29 ^c^	1.62 ^a^	0.25 ^c^	0.54 ^b^	0.122	<0.001
CHO, mM/L	2.09 ^b^	2.50 ^a^	2.27 ^ab^	2.50 ^a^	0.055	0.010
HDL-C, mM/L	1.01	1.07	0.95	1.06	0.022	0.181
LDL-C, mM/L	0.84 ^b^	1.38 ^a^	0.94 ^b^	1.06 ^b^	0.057	0.001
ALT, U/L	54.02 ^a^	44.42 ^b^	60.97 ^a^	59.28 ^a^	2.138	0.020
AST, U/L	29.32	28.53	31.41	29.52	1.135	0.853
TBIL, μM/L	3.59	3.35	3.73	3.23	0.159	0.695
ALP, U/L	134.67 ^b^	172.79 ^a^	141.11 ^b^	131.98 ^b^	5.554	0.035
CRE, μM/L	129.99 ^c^	160.46 ^a^	124.99 ^c^	144.83 ^b^	3.719	<0.001
Ca, mM/L	1.28 ^b^	1.25 ^b^	1.31 ^a^	1.26 ^b^	0.007	0.008
P, mM/L	2.66	2.38	2.63	2.53	0.075	0.601
TRF, mg/mL	0.24	0.24	0.26	0.25	0.017	0.980
HPT, μg/mL	1259.28 ^a^	983.37 ^b^	1260.69 ^a^	978.43 ^b^	51.190	0.042
CP, U/L	55.73	59.15	55.45	61.47	1.491	0.426

Values are presented as mean and pooled SEM, *n* = 6. Data in the same row with no or the same letter indicate no significant difference (*p* > 0.05), while different letters mean significant difference (*p* < 0.05). Abbreviations: NC, negative control; HITM, high-dose inorganic trace minerals; LITM, low-dose inorganic trace minerals; LOTM, low-dose organic trace minerals; GLU, glucose; TP, total protein; ALB, albumin; URE, urea; TG, triglyceride; CHO, cholesterol; HDL-C, high-density lipoprotein cholesterol; LDL-C, low-density lipoprotein cholesterol; ALT, alanine aminotransferase; AST, aspartate transaminase; TBIL, total bilirubin; ALP, alkaline phosphatase; CRE, creatinine; Ca, calcium; P, phosphorus; TRF, transferrin; HPT, haptoglobin; CP, ceruloplasmin.

**Table 9:** Effects of dietary replacement with high-dose inorganic trace minerals (Fe, Cu, Zn, Mn) with low-dose inorganic/organic alternatives on immunity function in growing-finishing pigs.

Items	NC	HITM	LITM	LOTM	SEM	*p*-Value
IgA, μg/mL	16.24	18.04	19.28	19.16	0.469	0.063
IgG, μg/mL	187.78 ^b^	217.01 ^ab^	247.59 ^a^	233.86 ^a^	7.487	0.019
IgM, μg/mL	18.33 ^b^	20.59 ^ab^	22.67 ^a^	22.90 ^a^	0.597	0.012

Values are presented as mean and pooled SEM, *n* = 6. Data in the same row with no or the same letter indicate no significant difference (*p* > 0.05), while different letters mean significant difference (*p* < 0.05). Abbreviations: NC, negative control; HITM, high-dose inorganic trace minerals; LITM, low-dose inorganic trace minerals; LOTM, low-dose organic trace minerals.

**Table 10:** Effects of dietary replacement of high-dose inorganic trace minerals (Fe, Cu, Zn, Mn) with low-dose inorganic/organic alternatives on nutrient apparent digestibility in growing-finishing pigs.

Items	NC	HITM	LITM	LOTM	SEM	*p*-Value
Energy, %	85.16 ^b^	88.92 ^a^	88.83 ^a^	88.90 ^a^	0.452	0.001
Dry matter, %	85.24 ^b^	88.90 ^a^	88.85 ^a^	88.92 ^a^	0.441	0.001
Crude protein, %	80.28 ^b^	85.30 ^a^	85.35 ^a^	86.21 ^a^	0.616	<0.001
Crude ash, %	39.08 ^b^	54.87 ^a^	53.89 ^a^	51.82 ^a^	1.677	<0.001

Values are presented as mean and pooled SEM, *n* = 6. Data in the same row with no or the same letter indicate no significant difference (*p* > 0.05), while different letters mean significant difference (*p* < 0.05). Abbreviations: NC, negative control; HITM, high-dose inorganic trace minerals; LITM, low-dose inorganic trace minerals; LOTM, low-dose organic trace minerals.

**Table 11:** Effects of dietary replacement of high-dose inorganic trace minerals (Fe, Cu, Zn, Mn) with low-dose inorganic/organic alternatives on tissue trace mineral distribution in growing-finishing pigs (mg/kg).

Items	NC	HITM	LITM	LOTM	SEM	*p*-Value
Serum						
Fe	0.62	1.33	1.50	1.33	0.149	0.158
Cu	1.87	2.34	2.12	2.22	0.078	0.180
Zn	1.11 ^b^	1.24 ^ab^	1.44 ^a^	1.50 ^a^	0.053	0.022
Mn	<0.01	<0.01	<0.01	<0.01	-	-
Liver						
Fe	106.77 ^b^	172.17 ^a^	136.50 ^ab^	152.67 ^a^	7.737	0.012
Cu	8.09 ^b^	12.82 ^a^	9.86 ^ab^	11.22 ^a^	0.587	0.024
Zn	80.24 ^b^	118.56 ^a^	89.37 ^b^	130.80 ^a^	6.147	0.003
Mn	3.61	3.79	3.63	3.57	0.102	0.890
Muscle						
Fe	3.65 ^b^	4.19 ^b^	4.32 ^b^	5.15 ^a^	0.171	0.009
Cu	0.495	0.457	0.469	0.496	0.012	0.614
Zn	15.93	15.47	15.50	17.00	0.345	0.378
Mn	0.013 ^d^	0.089 ^a^	0.029 ^c^	0.054 ^b^	0.006	<0.001

Values are presented as mean and pooled SEM, *n* = 6. Data in the same row with no or the same letter indicate no significant difference (*p* > 0.05), while different letters mean significant difference (*p* < 0.05). Abbreviations: NC, negative control; HITM, high-dose inorganic trace minerals; LITM, low-dose inorganic trace minerals; LOTM, low-dose organic trace minerals.

## Data Availability

The original contributions presented in this study are included in the article. Further inquiries can be directed to the corresponding authors.
